# Evaluation of Respiratory Symptoms Among Youth e-Cigarette Users

**DOI:** 10.1001/jamanetworkopen.2020.20671

**Published:** 2020-10-13

**Authors:** Alayna P. Tackett, Brittney Keller-Hamilton, Caitlin E. Smith, Emily T. Hébert, Jordan P. Metcalf, Lurdes Queimado, Elise M. Stevens, Samantha W. Wallace, Elizabeth L. McQuaid, Theodore L. Wagener

**Affiliations:** 1 Department of Preventive Medicine, Keck School of Medicine, University of Southern California, Los Angeles; 2 Center for Tobacco Research, The Ohio State University Comprehensive Cancer Center, Columbus; 3 Department of Psychology, Oklahoma State University, Stillwater; 4 School of Public Health, The University of Texas Health Science Center, Austin; 5 Department of Internal Medicine, University of Oklahoma Health Sciences Center, Oklahoma City; 6 Department of Otorhinolaryngology, University of Oklahoma Health Sciences Center, Oklahoma City; 7 T.H. Chan School of Public Health, Harvard University, Boston, Massachusetts; 8 Health Science Center, University of North Texas, Forth Worth; 9 Bradley/Hasbro Children’s Research Center, Providence, Rhode Island; 10 Department of Psychiatry and Human Behavior, Alpert Medical School of Brown University, Providence, Rhode Island; 11 Department of Internal Medicine, The Ohio State University, Columbus

## Abstract

**Importance:**

Use of e-cigarettes (ECs) among youths has increased in recent years. e-Cigarette aerosol contains chemical constituents, such as diacetyl or benzaldehyde, which are known to affect the respiratory system.

**Objective:**

To examine the association between EC use and self-reported wheezing in a cohort of US adolescents.

**Design, Setting, and Participants:**

This cohort study used data from waves 3 and 4 (October 19, 2015, to January 3, 2018) of the Population Assessment of Tobacco and Health (PATH) study, a longitudinal, nationally representative cohort survey. Adolescent respondents aged 12 to 17 years who did not have asthma were included.

**Exposures:**

e-Cigarette use during the previous year.

**Main Outcomes and Measures:**

Self-reported wheezing in the past 12 months (yes or no) and EC use (no use in past year or never use, use in past year, use in past 30 days, and use in past 7 days). Survey-weighted logistic regression models adjusted for demographic characteristics and other risk factors.

**Results:**

Among 7049 adolescents without asthma from waves 3 and 4 of the PATH study, 49.9% were female and 54.4% were non-Hispanic White. In unadjusted models, the odds of wheezing in the past 12 months were higher for youths who had used ECs in the past year compared with those who had not (odds ratio, 1.74; 95% CI, 1.22-2.48; *P* = .003). In the adjusted model, after controlling for the variables of race/ethnicity, household rules about the use of tobacco, contact with a smoker in the previous 7 days, and current use of combustible tobacco products, the association of EC use with wheezing was not significant (adjusted odds ratio for EC use in the past year, 1.37 [95% CI, 0.91-2.05]; in the past 30 days, 1.35 [95% CI, 0.63-2.88]; in the past 7 days, 0.74 [95% CI, 0.28-1.97]; *P* = .33).

**Conclusions and Relevance:**

In this cohort study, use of ECs alone was not associated with increased odds of experiencing wheezing episodes. Future studies incorporating the use of objective data appear to be needed to more accurately understand the potential respiratory harms associated with vaping among adolescents.

## Key Points

**Question:**

Is wheezing associated with e-cigarette use among youths?

**Findings:**

In this cohort study of 7049 adolescents in waves 3 and 4 of the Population Assessment of Tobacco and Health, the odds of wheezing in the past 12 months were higher for adolescents who had used e-cigarettes during the past year compared with those who had not used e-cigarettes. In the adjusted model, the association of e-cigarette use with wheezing was not significant.

**Meaning:**

The findings suggest that e-cigarette use alone might not be associated with increased odds of wheezing.

## Teaser

This cohort study uses data from the Population Assessment of Tobacco and Health from 2015 to 2018 to examine the association between wheezing and past-year use of e-cigarettes among youths.

## Introduction

Public health efforts have been effective in reducing the use of cigarettes among youths, and rates of cigarette use are the lowest ever recorded^1^; however, use of e-cigarettes (ECs) by youths has substantially increased in recent years.^1,2,3,4^ The findings from the 2018 National Youth Tobacco Survey showed a 75% increase in EC use among high school students between 2017 and 2018.^1,5^ This increased prevalence of EC use may be associated with the development of nicotine dependence and a future transition to other harmful tobacco products among adolescents.^6^ In addition, there may be respiratory effects of ECs; one concern is that the use of ECs may be associated with risk of respiratory symptoms such as wheezing among youths.^7,8^

Although the harmful respiratory effects of combustible tobacco use are well documented,^9,10,11,12^ little is known about the respiratory effects of EC use. e-Cigarette aerosol contains several chemical constituents, such as diacetyl or benzaldehyde, that affect the respiratory system.^13,14,15,16,17^ To date, only a few studies have examined the respiratory outcomes associated with EC use in humans.^8,18,19,20,21^ The data indicate an association between adolescent EC use, bronchitis symptoms,^8^ and increased cough or production of phlegm.^18^

Studies specifically examining the association between respiratory symptoms, such as wheezing, and EC use among adolescents are needed to identify preliminary associations between EC use and respiratory symptoms. A majority of the literature is focused on use measured at 1 time, such as ever or in the past 30 days, and limited data have examined whether long-term EC use is associated with increased risk for adverse respiratory effects in adolescents. Therefore, with use of public data from the Population Assessment of Tobacco and Health (PATH), this cohort study examined whether a history of EC use, combustible tobacco co-use, demographics, and household characteristics are associated with the prevalence of wheezing among youths.

## Methods

### Setting and Procedures

This cohort study used publicly available data from the PATH study, an ongoing, nationally representative, longitudinal cohort study of adolescents and adults in the US. The PATH study uses audio, computer-assisted self-interviews, available in English and Spanish, to collect self-reported information on tobacco use patterns and associated health behaviors. Four waves of data collection have been completed, and the present analysis focuses on waves 3 and 4. Wave 3 data collection occurred from October 19, 2015, to October 23, 2016, and wave 4 data collection was conducted from December 1, 2016, to January 3, 2018. The PATH study recruitment used a stratified, address-based, area-probability sampling design during wave 1. An in-person screener was used during wave 1 to select adolescents and adults from households for participation. Details about survey interview procedures, questionnaires, sampling, weighting, and accessing the data are available elsewhere.^22^ The PATH study is conducted by Westat and was approved by the Westat institutional review board. All youth participants aged 12 to 17 years in the PATH study provided assent, and their parent or legal guardian provided consent. This study followed the Strengthening the Reporting of Observational Studies in Epidemiology (STROBE) reporting guideline.

At wave 1, the weighted response rate for the household screener was 54.0%. Among households that were screened, the overall weighted response rate for the adolescent interview was 78.4% at wave 1, 87.3% at wave 2, 83.3% at wave 3, and 79.5% at wave 4. Interviews were completed with 13 651 adolescents (aged 12 to 17 years) at wave 1, 12 172 at wave 2, 11 814 at wave 3, and 11 059 at wave 4. The differences in the number of completed interviews between wave 1 and later waves reflect attrition owing to nonresponse, aging out of the sample, mortality, replenishment sampling, and other factors. The analyses in this study included participants who provided pertinent data for waves 3 and 4, were younger than 18 years at wave 4, and did not have a diagnosis of asthma.

### Measures

#### Outcome Variable

The outcome variable was any episode of wheezing in the past 12 months (yes or no) as reported on the wave 4 survey. For participants who completed the wave 3 survey (ie, participants whose data were used in this analysis), wheezing was assessed by asking, “Have you had wheezing or whistling in the chest in the past 12 months?”

#### Exposure Variable

We constructed a 4-level measure describing EC use at wave 3 as follows: (1) no use in the past year or never use, (2) use in the past year, (3) use in the past 30 days, and (4) use in the past 7 days. Participants were first asked whether they had ever used an EC, even if only once or twice. Those who reported ever trying an EC were asked to report the last time that they used one.

#### Covariates

Covariates included demographic variables and variables identified as potential confounders of the association between EC use and wheezing. Demographic variables included self-reported sex (male or female), race/ethnicity (non-Hispanic White, non-Hispanic Black, non-Hispanic other, or Hispanic), age category (12-14 years or 15-17 years), and household income (<$25 000, $25 000 to<$50 000, $50 000 to <$100 000, or ≥$100 000). Sex and race/ethnicity data from wave 1 were used. Age categories and household income data from wave 3 were used.

Confounding variables were identified by testing the variables’ associations with EC use and wheezing during the past 12 months; variables that were associated with both at the α = .05 level of significance were included in the multivariable models. These variables included rules about the use of combustible and noncombustible tobacco products in the home (never allowed vs sometimes or always allowed), use of combustible tobacco products in the past 30 days (any vs none; products included cigarettes, traditional cigars, cigarillos, filtered cigars, pipes, hookahs, bidis, and kreteks), and time spent in close contact with a smoker in the past 7 days (0 hours vs ≥1 hour). Other variables that were tested but did not meet the definition of confounding included self-rated health and living with a tobacco user. Confounding variable data from wave 3 were used.

### Statistical Analysis

Wave 4 all-waves weights were used in all analyses to account for differing probabilities of selection, nonresponse, and study attrition. Standard errors were calculated using replicate weights that were computed using the Fay balanced repeated replication method.^23^ The domain (ie, subpopulation) specified for all analyses was adolescents without asthma who had complete data on EC use at wave 3 and on wheezing at wave 4. Adolescents with asthma were excluded because of their small sample size; their inclusion in the analysis would lead to imprecise point estimates. Analyses were conducted using svy procedures in Stata/SE, version 16.0 (StataCorp LLC). Missing data on sex, race/ethnicity, and age were single-imputed by the PATH study team.^22^

First, descriptive statistics were calculated to evaluate the distributions of demographic and confounding variables according to the EC user group. Rao-Scott χ^2^ tests of the associations between these covariates and the EC user groups were also conducted. Second, unadjusted associations between EC user groups and wheezing during the past 12 months (yes or no) were modeled using logistic regression. Third, demographic covariates and other confounders described above were added to multivariable logistic regression models to produce adjusted estimates. Statistical significance was assessed at α = .05.

## Results

### Participant Characteristics

Among 7049 adolescents without asthma from waves 3 and 4 of the PATH study, 49.9% were female and 54.4% were non-Hispanic White. A total of 6438 adolescents (91.3%) had not used an EC in the past 12 months at wave 3 (Table 1). With the exception of sex and household income, adolescent characteristics differed across EC user groups (Table 1). Notable differences among EC user groups included the following: (1) higher probability of being non-Hispanic White among those who had used ECs during the past 30 days and past 7 days, (2) higher probability of combustible or noncombustible tobacco use permitted in the home (ie, sometimes or always) among adolescents who had used ECs in the past year or more recently, (3) higher probability of spending at least 1 hour in close contact with a smoker in the past 7 days among adolescents who had used ECs in the past year or more recently, and (4) higher probability of using a combustible tobacco product in the past 30 days among adolescents who had used ECs in the past year or more recently.

**Table 1.** Demographic Characteristics, Risk Factors, and Wheezing Associated With e-Cigarette Use Among Adolescents Without Asthma^a^

Characteristic	Most recent use of an e-cigarette, %^b^				
	Never or >1 y ago (N = 6438)	Past year (N = 417)	Past 30 d (N = 95)	Past 7 d (N = 99)	*P* value^c^
Sex					
Female	50.2	46.7	52.7	40.8	.22
Male	49.8	53.3	47.3	59.2	
Annual household income					
<$25 000	21.5	25.1	20.2	26.1	.08
$25 000 to<$50 000	21.3	20.6	26.1	25.3	
$50 000 to<$100 000	27.3	32.6	21.2	24.6	
≥$100 000	29.9	21.6	32.6	24.0	
Race/ethnicity					
Non-Hispanic White	53.9	57.6	68.2	61.7	.04
Non-Hispanic Black	12.8	6.8	5.8	9.5	
Non-Hispanic other^d^	9.8	9.8	4.2	7.7	
Hispanic	23.5	25.8	21.8	21.1	
Age category					
12-14 y	64.0	31.2	30.7	27.0	<.001
15-17 y	36.0	68.8	69.3	73.0	
Rules about tobacco use in the home					
Never allowed	80.0	62.2	70.5	62.1	<.001
Sometimes or always allowed	20.0	37.8	29.5	37.9	
Combustible tobacco use in past 30 d^e^					
No	99.2	82.3	73.2	58.8	<.001
Yes	0.8	17.7	26.8	41.2	
Time in close contact with a smoker in past 7 d					
0 h	73.8	43.6	33.6	33.5	<.001
≥1 h	26.2	56.4	66.4	66.5	
Wheezing in past 12 mo at wave 4					
No	92.4	87.5	88.0	90.3	.005
Yes	7.6	12.5	12.0	9.7	

^a^ Data are from the Population Assessment of Tobacco and Health, waves 3 and 4 (October 2015 to January 2018). Percentages were survey weighted to account for the complex sampling design; unweighted participant counts are reported. The analytic domain included participants without asthma who had complete data on e-cigarette use at wave 3 and complete data on wheezing at wave 4. Percentages may not sum to 100 owing to rounding.

^b^ e-Cigarette use was assessed at wave 3. Categories are mutually exclusive.

^c^ *P* values were calculated using Rao-Scott χ^2^ tests.

^d^ Non-Hispanic other included American Indian or Alaskan Native, Asian Indian, Chinese, Filipino, Japanese, Korean, Vietnamese, other Asian, Native Hawaiian, Guamanian or Chamorro, Samoan, and other Pacific Islander.

^e^ Combustible tobacco products included cigarettes, traditional cigars, cigarillos, filtered cigars, pipes, hookahs, bidis, and kreteks.

### Association of Wheezing With EC Use

At wave 4, 568 of 7049 participants (8.1%) in the analytic domain reported wheezing in the past 12 months. Use of ECs was associated with odds of any wheezing in the past 12 months in the unadjusted model (odds ratio [OR] for EC use in the past year, 1.74 [95% CI, 1.22-2.48]; in the past 30 days, 1.66 [95% CI, 0.86-3.21]; in the past 7 days, 1.31 [95% CI, 0.63-2.69]) (Table 2). Point estimates for ORs comparing each EC user group with the reference group of adolescents who had not used ECs in the past year were statistically similar, although only those who had used ECs in the past year had significantly increased odds of wheezing (OR, 1.74; 95% CI, 1.22-2.48). In the adjusted model (Table 2), the association of EC use with wheezing was not significant (OR for EC use in the past year, 1.37 [95% CI, 0.91-2.05]; in the past 30 days, 1.35 [95% CI, 0.63-2.88]; in the past 7 days, 0.74 [95% CI, 0.28-1.97]). Only time spent in close contact with a smoker in the past 7 days was associated with wheezing in the past 12 months in the multivariable model (OR, 1.54; 95% CI, 1.22-1.95; *P* < .001).

**Table 2.** Odds of Wave 4 Wheezing According to Wave 3 e-Cigarette Use Among Adolescents Without Asthma^a^

Characteristic	Any wheezing in past 12 mo, OR (95% CI)	*P* value^**b**^
**Unadjusted model results**		
e-Cigarette use^c^		
Never or >1 y ago	1 [Reference]	NA
Past year	1.74 (1.22-2.48)	.003
Past 30 d	1.66 (0.86-3.21)	.13
Past 7 d	1.31 (0.63-2.69)	.47
**Adjusted model results**		
e-Cigarette use^c^		
Never or >1 y ago	1 [Reference]	NA
Past year	1.37 (0.91-2.05)	.13
Past 30 d	1.35 (0.63-2.88)	.43
Past 7 d	0.74 (0.28-1.97)	.54
Sex		
Male	1 [Reference]	NA
Female	1.12 (0.91-1.38)	.27
Household income		
<$25 000	1 [Reference]	NA
$25 000 to<$50 000	0.89 (0.66-1.19)	.44
$50 000 to<$100 000	1.04 (0.77-1.41)	.78
≥$100 000	1.04 (0.78-1.49)	.79
Race/ethnicity		
Non-Hispanic White	1[Reference]	NA
Non-Hispanic Black	1.09 (0.77-1.55)	.63
Non-Hispanic other^d^	0.86 (0.60-1.23)	.40
Hispanic	0.86 (0.63-1.15)	.30
Age category, y		
12-14	1 [Reference]	NA
15-17	0.95 (0.77-1.18)	.66
Household ban on tobacco use		
Never allowed	1 [Reference]	NA
Sometimes or always allowed	1.25 (0.97-1.62)	.08
Combustible tobacco use in past 30 d^e^		
No	1 [Reference]	NA
Yes	1.21 (0.65-2.25)	.54
Time in close contact with a smoker in past 7 d, h		
0	1 [Reference]	NA
≥1	1.54 (1.22, 1.95)	<.001

Abbreviations: NA, not applicable; OR, odds ratio.

^a^ Data are from the Population Assessment of Tobacco and Health, waves 3 and 4 (October 2015 to January 2018). All results were survey weighted to account for the complex sampling design. The analytic domain included participants without asthma who had complete data on e-cigarette use at wave 3 and complete data on wheezing at wave 4.

^b^ *P* values were calculated using Wald tests.

^c^ Use of e-cigarettes was assessed at wave 3. Categories are mutually exclusive.

^d^ Non-Hispanic other included American Indian or Alaskan Native, Asian Indian, Chinese, Filipino, Japanese, Korean, Vietnamese, other Asian, Native Hawaiian, Guamanian or Chamorro, Samoan, and other Pacific Islander.

^e^ Combustible tobacco products included cigarettes, traditional cigars, cigarillos, filtered cigars, pipes, hookahs, bidis, and kreteks.

## Discussion

In unadjusted models, we identified a statistically significant association between EC use (measured at wave 3) and increased odds of any wheezing in the past 12 months (measured at wave 4) in a nationally representative sample of adolescents. However, after adjusting for demographic factors and other confounding variables, this association was not significant. These results suggest that having close contact with current smokers may be more strongly associated with the occurrence of wheezing in the past month than EC use, as operationalized in the current study.

Limited data are available regarding respiratory symptoms among adolescent users of ECs, and, to our knowledge, this is the first population-based, longitudinal analysis of the association between EC use and wheezing among adolescent respondents. Previous literature suggests that bronchitis symptoms (chronic cough, phlegm production, or bronchitis) are associated with the frequency of adolescent EC use.^8,18^ However, the findings of the current study appear to be consistent with recent work suggesting that covariates (eg, secondhand smoke exposure) account for part of the association between EC use and wheezing episodes.^8^ McConnell and colleagues^8^ examined the association of EC use with self-reported wheezing; however, after controlling for use of combustible tobacco, exposure to secondhand smoke, and past use of ECs, this association was no longer significant. This consistency in findings may be best explained by methodologic commonalities. Specifically, both studies relied on self-reported wheezing symptoms, which are subject to recall bias. In addition, owing to small data cells, the present study excluded adolescents with asthma. Adolescence is a dynamic developmental period of physiologic changes, including an increase in lung function and volume.^24,25^ Little is known regarding the specific cause and categorization of adolescent wheezing.^26^ One study^26^ of healthy adolescents with no asthma found that adolescents who presented with undiagnosed wheeze had increased odds and earlier onset of combustible tobacco smoking,^26^ rhinitis,^26^ and a family history of asthma,^26^ suggesting that undiagnosed wheeze differs from diagnosed asthma.^26^ Future research, including longitudinal methods incorporating objective data and comparative studies of youths with and without asthma, are needed to more accurately understand the respiratory risks associated with EC use among youths.

Results from this study build on previous findings in the literature.^8,12,19,27,28^ A longitudinal secondary data analysis^12^ using the adult PATH data reported that EC use was an independent risk factor associated with respiratory disease (eg, chronic obstructive pulmonary disease) and that adults using dual products (EC and combustible tobacco) had significantly increased odds of respiratory disease compared with those using only ECs. Another study^29^ using cross-sectional data from the adult wave of PATH (wave 2) reported an increased risk of wheezing and related respiratory symptoms among exclusive EC users, exclusive smokers, and dual users. Similar to findings of the longitudinal study, exclusive EC users experienced increased risk for wheezing and related respiratory symptoms, but the risk was lower than respiratory risks for current smokers or dual users. The findings of the current study suggest that the increased odds of experiencing wheezing within the past 12 months among adolescents may be associated with other variables and not with EC use specifically. Factors associated with negative respiratory symptoms may differ depending on age; however, future studies are needed to assess which demographic characteristics have the strongest association with these symptoms.

With regard to covariates associated with history of EC use in this sample, adolescents who had used ECs in the past 30 days and/or the past 7 days had a higher probability of being non-Hispanic White. This finding is similar to other studies^30,31^ that showed that non-Hispanic White adolescents had increased odds of EC use. Adolescents who had used ECs in the past year or less had an increased probability of combustible or noncombustible tobacco use permitted in the home. Many studies have highlighted associations between a household ban and initiation of tobacco or EC use^32,33,34,35^ as well as harms related to secondhand smoke exposure,^32,34,35,36^ which is also associated with adolescent respiratory health and symptoms. In the present study, adolescents who had used ECs in the past year or less also had an increased probability of spending at least 1 hour in close contact with a smoker in the past 7 days and using a combustible tobacco product in the past 30 days. This finding is consistent with other emerging research highlighting that use of multiple tobacco products may be common among adolescents in the US.^37^ In addition, other research examining adolescent use of ECs indicated that EC use may be associated with risk of transition to other products, such as combustible cigarettes, among adolescents.^7,38,39,40^ Taken together, these findings highlight the complexity of examining EC use and respiratory symptoms among adolescents. Patterns of EC use (ever vs current, long-term use), peer and caregiver exposure, and co-use of other products (eg, combustible tobacco) may exacerbate respiratory risks associated with EC use in this age demographic and should be considered.

### Strengths and Limitations

This study has strengths. The study used the nationally representative PATH data with a large sample size of adolescent respondents. This study also has limitations. First, the PATH data are self-reported and may be subject to recall bias. Adolescent respondents were asked to recall symptoms of wheezing during the past year, and thus, these data may be an inaccurate reflection of symptoms. Second, the frequency of EC use in the past 30 days among adolescent respondents in the PATH study (1.5%) is substantially lower than the current national average (20%).^1^ Although the present data are longitudinal, they may not be reflective of actual use patterns to date. For example, from 2017 to 2018 during the increase of pod-mod ECs (eg, salt-based, pod-system products such as JUUL), there was a 78% increase in use among high school students and a 48% increase among middle school students.^41,42^ The EC data in this study were collected in 2015 and 2016 and thus did not include a systematic assessment of use of pod-mod–style ECs. Nicotine-salt EC devices allow for higher nicotine concentrations in e-liquids and may be associated with higher mean flow rates and puff volumes.^43^ Therefore, these analyses should be replicated with data that include nicotine-salt EC devices. Third, although we examined EC use and important covariates temporally before we examined wheezing, these analyses cannot provide evidence for the cause-and-effect relationship between EC use and wheezing. In addition, the PATH study provides limited questions to adolescent respondents regarding respiratory symptoms (eg, shortness of breath is only asked of respondents with asthma) and does not incorporate objective measures of pulmonary health (eg, spirometry); these data are important to incorporate into future studies examining associations of respiratory symptoms with EC use among adolescents.

## Conclusions

In this cohort study, use of ECs alone was not associated with an increased risk of wheezing among adolescents when other risk factors for respiratory symptoms were controlled. The findings suggest that other risk factors, including secondhand smoke exposure, may be associated with the development of negative respiratory symptoms among adolescents. Future studies that include a larger number of EC users, incorporate objective measures of respiratory physiology, and more comprehensively assess respiratory symptoms appear to be needed to better understand these associations and delineate risk patterns among adolescents.
